# Patient experience of follow‐up after surgery for kidney cancer: a focus group study

**DOI:** 10.1111/bju.15982

**Published:** 2023-02-22

**Authors:** Hannah Harrison, Grant D. Stewart, Juliet A. Usher‐Smith

**Affiliations:** ^1^ Department of Public Health and Primary Care University of Cambridge Cambridge UK; ^2^ Department of Surgery University of Cambridge, Addenbrooke's Hospital Cambridge UK

**Keywords:** kidney cancer, follow‐up, surveillance, patient experience, focus group study, #kcsm, #KidneyCancer, #uroonc

## Abstract

**Objective:**

To explore patient experience of follow‐up care after kidney cancer surgery and to develop recommendations for best practice.

**Methods:**

We conducted two focus groups, including 14 participants with experience of kidney cancer follow‐up after surgery, to elicit patient views on current follow‐up care. Thematic analysis was used to identify unifying themes to describe the patient experience of follow‐up, and the results were then used to develop a set of recommendations for best practice.

**Results:**

We identified six themes (feelings of abandonment; uncertainty about the plan; anxiety about appointments; variation in care; a need for information; and a need for emotional support) that described current patient experience and areas in which current care could be improved. In particular, while most of the participants felt that their physical needs had been met, many had struggled with unmet emotional needs and a lack of information and resources. This was especially noted in the period immediately following surgery, when feelings of abandonment were common, and around follow‐up scans and routine appointments, which were a source of anxiety. Our participants also described concerns about the lack of consistency between different hospitals and centres around the United Kingdom, with differences in the content and quality of follow‐up care. Based on the results, we developed a list of recommendations to address some of the challenges described through relatively minor changes to the care pathway.

**Conclusions:**

We identified gaps and variability in current follow‐up care after kidney cancer surgery, and have developed a set of recommendations that, if implemented, would improve the follow‐up care experience for these patients.

## Introduction

Approximately one in five patients who have surgery for presumed localized RCC (subsequently referred to as kidney cancer) go on to develop incurable metastatic disease [1]. The management of localized kidney cancer, including follow‐up, is carried out by a multispecialty team [2]. In line with current European guidelines, all patients undergo a long surveillance programme (5–10 years) including regular imaging following nephrectomy, with the aim of detecting recurrent disease early [3, 4]. Aspects of the follow‐up schedule, including the frequency of scans, are stratified by the risk of recurrence for each patient. However, there is significant variation in the clinical management of these patients after surgery; the choice of method for risk stratification (for example, Leibovich score) is left to individual clinicians [4] and follow‐up schedules are heterogeneous [5].

A survey showed that many patients with kidney cancer experience distress (67%) and feared cancer recurrence (55%), with this affecting their quality of life and health outcomes [6]. In a UK survey of patients undergoing surveillance following kidney cancer surgery, 85% of respondents had increased levels of anxiety due to delays in receiving results, 48% were not confident that future results would be shared in a timely manner and 43% had ‘chased’ before receiving the results of their latest scan [7]. Improvements to kidney cancer follow‐up care and a more personalized approach have been identified as priorities by patients [8]. Research investigating the views and experiences of patients for other cancers have identified gaps in follow‐up care [9, 10, 11]. However, no in‐depth studies have explored patient experiences following kidney cancer.

## Methods

### Study Design

Qualitative research methods allow the in‐depth exploration of the experiences and perspectives of the research participants. In focus groups, participants have the opportunity to build on the responses of others.

We conducted two focus groups with patients who had experience of kidney cancer follow‐up care after surgery. Ethical approval was provided by the University of Cambridge Psychology Research Ethics Committee (PRE.2021.082).

### Research Team

The research team consisted of three researchers with different expertise: a public health researcher, an academic GP and a urology consultant. Two patients with experience of kidney cancer follow‐up were involved throughout the study.

### Participants

Recruitment for the focus groups was carried out through the charity Kidney Cancer UK (KCUK). An advertisement (Appendix S1) was posted in two KCUK Facebook groups (combined membership of 4500). The advert was also posted on the KCUK Twitter feed (2000 followers) and placed in a newsletter distributed to 700 people with a history of kidney cancer. Eligible individuals (adults living in the United Kingdom with previous surgery for kidney cancer), were put in contact with the research team and given a participant information sheet (Appendix S2). Before joining a focus group, the research team obtained informed consent (via an electronic form or telephone) from all participants. None of the participants was known to the research team prior to study recruitment.

### Focus Groups

The two focus groups were held in January 2022 using Zoom video conferencing software. The groups were held on consecutive weekdays, the first in the afternoon and the second in the evening. Prior to attending the focus groups, participants completed a short questionnaire, which included basic demographic information and the follow‐up they had received (Appendix S3).

Each focus group lasted approximately 1 h and was facilitated by two members of the research team. H.H., a research associate with experience in public health research and training in qualitative methods, chaired both sessions and guided the discussion through a series of topics relating to follow‐up care (Appendix S4). The discussion covered follow‐up care immediately after kidney cancer surgery and long‐term surveillance for recurrent disease. J.U.S., an academic GP with experience in qualitative research, observed the sessions, monitored the chat and provided any non‐clinical support required by the participants.

### Analysis

Video recordings of the focus groups from Zoom were transcribed by an external company; all identifying information was removed during transcription. The transcripts were then analysed using thematic analysis.

We familiarized ourselves with the data by watching the video recordings and reading through the transcripts. A provisional coding framework was developed by H.H. based on her initial impressions of the discussions. The transcripts were then coded in nvivo12 software by both H.H. and J.U.S., and the coding framework was iteratively revised and refined through discussion between the researchers. After coding was complete, the codes were grouped into themes describing unifying concepts. The themes were reviewed by the whole research team, and the coding of the two researchers was compared. Quantitative data from the questionnaires was extracted by H.H. and analysed descriptively. The results were checked for validity by two patient representatives and a patient trustee working for KCUK.

## Results

Fourteen participants attended the focus groups (six and eight, respectively). More than half of the attendees were women (*n* = 8) and all self‐reported White ethnicity. The participants were aged between 40 and 80 years, with most aged under 60 years (*n* = 10). Eleven of the participants were <2 years post‐surgery, and one was more than 5 years post‐surgery. Nearly all of the participants (*n* = 12) reported attending at least one follow‐up appointment (Table 1).

**Table 1:** Characteristics of study participants.

Characteristics	Number	Percentage (%)
Gender		
Men	6	42.9
Women	8	57.1
Age group		
<40 years	0	0.0
40–49 years	5	35.7
50–59 years	5	35.7
60–69 years	3	21.4
70–79 years	1	7.1
>79 years	0	0.0
Time since first diagnosis		
<1 year	6	42.9
1–2 years	5	35.7
2–5 years	2	14.3
>5 years	1	7.1
Attended follow‐up appointments		
Yes	12	85.7
No	2	14.3

Six themes, listed in Table 2 alongside representative quotations, were identified that are key to the follow‐up experiences of the focus group participants. The final coding framework can be found in Appendix S5.

**Table 2:** Themes and illustrative quotations.

Theme	Illustrative quotation
Abandonment	‘And I wanted to be part of this because, although the surgery went really well, I do feel as I have been left a little bit to my devices.’ (P4, FG2) ‘…at the time I just sort of felt sort of tossed into the wilderness after my operation…’ (P3, FG1) ‘I've been left in a lot of pain and very little support or follow up; so it sounds a very similar story.’ (P2, FG2)
Uncertainty about the plan	‘Mine was clearly explained to me on the phone and in a letter exactly what was we were going to be doing for the next 2 years and he said it was because it was a particularly aggressive cancer, so they did explain all of that to me on the phone and in a letter as well.’ (P4, FG2) ‘I guess I received a letter explaining but then some of the terminology in the letter I just did not understand, and nobody really explained it to me even when I asked initially.’ (P8, FG2) ‘He said to me the likelihood was being very high risk we would reckon 85% of people would see some reoccurrence of cancer somewhere else in the body in the next 5 years. But he just threw that out there and it was… You do not have time to react’ (P4, FG1)
Anxiety about follow‐up	‘I think the thing I found hardest with all of it was just that expectation management, so if they said 6 months and it is seven I would find that really difficult.’ (P5, FG2) ‘I always had to chase the fact that I was going to have a follow‐up scan…’ (P3, FG1) ‘And for the 2 weeks or 3 weeks afterwards [after the scan] you are left thinking any time that phone rings it is going to be an oncologist telling me it is back.’ (P4, FG1) ‘I think it is one of the… For me personally I think it is one of the worst things is just waiting for those results all the time, it's just…’ (P3, FG1) ‘Yeah’ (P5, FG1) ‘…and it's not so bad now because I'm under Oncology and they see my quite quickly and they tell me quite quickly. But I know certainly in the beginning it did take a long time.’ (P3, FG1) ‘Yeah, I agree with you there it does bother me, obviously I'm keen to know if this other one [tumour] is cancerous or not, so this next one is already starting to tick at my brain that is coming up.’ (P1, FG1)
Variation in care	‘…because there's a real disparity from one end of the United Kingdom to the other in terms of what we get as after care…’ (P4, FG1) ‘I think if you sort of live under one of the like specialisthThe evidence beyond the guidelin kidney cancer places you get a lot more information and a lot more help and things. If you sort of live somewhere that is not I think… you are very much on your own to your extent a lot of the time’ (P3, FG1) ‘If they are going to use the Leibovich score then everyone uses the Leibovich score. If some are and some aren't then they should not use it at all, they should not give it to people.’ (P4, FG1) ‘Do any of you talk to your consultant regularly? I talk to my consultant every month and through Covid; I've been to the hospital every month because I have to go. Results are face to face even through Covid, she will not give you a result unless it is face to face. […]’ (W3) ‘Well, that makes me feel like I am living on a different planet because I've seen my consultant once and that was at the 6 week review [when] I complained of abdominal pain…’ (P2, FG2)
Need for information	‘How do you live with one kidney? I did not really get too much explanation it has been a bit hit and miss….And I've just had to do my own research and try and work out what is there.’ (P3, FG2) ‘At my 6 week appointment when I was told everything that it was cancer and that I would have annual scans he mentioned ultrasound or it might be CT. So, I sort of thought okay at the time I was so blown away that it was cancer so it was hard to take it in…’ (P1, FG1) ‘I think I would have been scared to death if he [the consultant] had told me 85% [survival] chance in the next 5 years… I want to be educated but at the same time I want to be a bit oblivious.’ (P6, FG1)
Emotional support	‘I like to see people's stories [on KCUK online group] when they are better, I find that really helpful and uplifting sort of thing.’ (P3, FG1) ‘I do too.’ (P1, FG1) ‘I think it's the double edge sword is not it? It is good to see the good ones but part of my counselling there was a story that they put on there about a daughter who was at university and she made a video of her mum. Her mum had gone in and had all…pretty much exactly the same as I had and here it was really bad and she had got and it was now terminal.’ (P6, FG1) ‘I suppose I find the surgical teams and the oncology teams they are medical fixers and they do that… but the gaps around the emotional support and how you cope with that…I have had a mixture of emotions where I've been at a very dark place and there are days when I'm very positive. Someone mentioned Macmillan I think that's…Yeah I certainly think Macmillan are very useful and at one point I got involved in some counselling just trying to help me come to terms with it…’ (P3, FG2)

FG, focus group; P, patient.

### Feelings of Abandonment

While some participants were positive about the transition from active treatment to follow‐up care ‘*I feel like my care has been really good and I have been really well supported*’ (patient [P]6, focus group [FG]1), many others described feeling abandoned after their surgery, as they transitioned into follow‐up care. This was described in contrast to the busy and stressful period during diagnosis and leading up to surgery, during which they had had a lot of contact with the hospital and clinicians. The sudden lack of contact, especially during this period of recovery from surgery, left a lasting impression, with participant descriptions of this ranging from ‘*left a little bit to my own devices*’ (P4, FG2), to ‘*tossed into the wilderness’* (P3, FG1) or going into a *‘black hole’* (P6, FG2).

When talking about feeling alone after surgery, several participants mentioned feeling unsure about the next steps in their care pathway and underprepared to manage aspects of their aftercare, and felt they would have benefitted from more support. In particular, some participants described being ‘*left in a lot of pain and very little support or follow‐up’* (P2, FG2), while others had felt unprepared for aspects of wound care and the side effects of the surgery, and did not know whom they should contact with any questions.

Similarly, participants had concerns about other transitions in their care pathway, including moving between departments (for example, Urology to Oncology) or moving back to primary care from secondary care. In these discussions, some patients reported feeling that it was difficult to ‘*keep in the loop*’ (P7, FG2) or that information was not always passed quickly and accurately between clinical teams or departments.

### Uncertainty about the Plan

There was variation in how well the participants felt they understood their care plan for long‐term follow‐up. Most participants felt that their follow‐up plan had been well explained, and understood why they would be having more (or less) surveillance than other patients in the context of their personal risk of recurrence. These participants positively described being given clear outlines of their care going forward, including the treatment options available in case of recurrent disease.

This was not, however, a universal experience, with other participants describing receiving letters with terminology they did not understand, or not understanding why the frequency of imaging had reduced over time. One participant, for example, described receiving a different type of imaging at one of her appointments (a chest X‐ray instead of a CT scan) with no explanation.

Participants expressed more confidence in their plan, and how it related to their individual risk of recurrence, when they had received both a verbal and written explanation *‘Mine was clearly explained to me on the phone and in a letter exactly what it was we were going to be doing for the next two years’* (P4, FG2). When participants received information of this type only by verbal explanation, they described feeling that they did not have ‘*time to react*’ and that they ‘*forget everything that has been said to me*’ (P4, FG1) after the appointment.

Some participants were also concerned about what was going to happen after their follow‐up ended. These participants perceived their risk of recurrence as remaining high and were concerned about metastatic disease being missed. One attendee described being given their 5‐year survival rate, and feeling that this meant they were ‘*only going to last five years*’ and that there was ‘*no plan*’ (P4, FG2) after this point.

### Anxiety about Appointments

Anxiety about follow‐up appointments was a universal experience of the focus group attendees, with concerns about the scheduling of appointments and waiting for the results of scans discussed at length.

Some participants said that they ‘*always had to chase*’ (P3, FG1) to book follow‐up scans and that uncertainty around scheduling increased their anxiety. For some participants, this was linked to expectation management, with one patient explaining ‘*if they said six months and it is seven* [months] *I would find that really difficult*’ (P5, FG2). Another participant described being reassured by having the next scan booked immediately following her previous appointment.

Most of the participants found the time after a scan before they received the results challenging, and several specifically identified this as ‘*scanxiety*’. Attendees described this as ‘*horrendous*’ and ‘*one of the worst things*’ (P3, FG1) and that they felt ‘*constantly*’ (P6, FG1) anxious as they waited for results. While participants disliked waiting several weeks for a scheduled appointment to discuss their results, this seemed to be preferable to waiting for unscheduled phone appointments. One participant described spending weeks ‘*thinking any time the phone rings it's going to be an oncologist telling me it* [kidney cancer] *is back*’ (P4, FG1). Again, expectation management was important, with one patient saying that waiting 6 weeks was ‘*really hard to deal with*’ (P5, FG2) after they had been told to expect results within 3 weeks.

### Variation in Care

A clear concern of the participants in this study was the difference between their care and that of others, perceived as a ‘*postcode lottery*’ (P5, FG1). Based on discussions with other patients, both before and during the focus groups, participants felt that ‘*there's a real disparity from one end of the United Kingdom to the other*’ (P4, FG1) in both the amount and the quality of care. This included individuals who felt that their care had been good, but that this was not true for all patients, and those who felt they had received substandard care.

Specific areas of variation mentioned included: the frequency with which patients were seen by clinicians; the availability of an assigned contact (such as a specialist nurse or consultant); and the amount of information provided about follow‐up care. Some patients highlighted that they felt that ‘*a lot more information and a lot more help*’ (P3, FG1) was available for people who attended specialist kidney cancer centres.

Participants were also aware that different follow‐up schedules were used by different centres, including different scan frequencies and imaging types being used for people with similar levels of risk. There was consensus that care should be standardized and that all patients should be offered the same care regardless of where they were treated. This included the methods to determine risk of recurrence: ‘*If they are going to use the Leibovich score then everyone uses the Leibovich score. If some are and some aren't then they shouldn't use it at all, they shouldn't give it to people*’ (P4, FG1).

### A Need for Information

During the sessions, participants spent considerable time discussing what information had been provided about follow‐up care, how this had been communicated and what information they would like to have been given.

Participants specifically mentioned feeling unprepared for pain management and wound care, and said they would like to have information on these areas. After surgery, several people felt that they needed more information about ‘*living with one kidney*’ (P3, FG2); discussion revealed uncertainty around the guidelines for alcohol consumption and ibuprofen use, as well as how reduced kidney function would affect day‐to‐day life. For example, one participant described being giving ibuprofen in hospital after their surgery, despite also receiving advice not to take ibuprofen.

For most participants, the longest conversation about their long‐term follow‐up care was during an appointment with a consultant approximately 6 weeks after surgery. Several participants felt that this appointment had gone well, and that a clear explanation of their ongoing surveillance for recurrent disease and the clinical justification for this had been delivered. However, this 6‐week appointment is also used as a debrief covering the outcome of the surgery itself, and patients who did not have a biopsy before surgery have their diagnosis of cancer confirmed. A participant with this experience, recalled that after receiving this information, they were ‘*blown away*’ and it was ‘*hard to take in*’ (P1, FG1) any other information. Receiving a written explanation after the appointment that repeated the follow‐up care plan and the justification for this plan was mentioned positively.

There were, however, differing perspectives between the participants with respect to how much information they wanted, especially about their risk of recurrent disease and survival odds. Several people said that it had been helpful to see their scan results, while others were frustrated that this was not offered and some were hearing for the first time that this was possible. One participant felt that if risk was going to be discussed, it was essential that a very clear explanation be given, ‘*not just kind of throw science at me and then not explain*’, in order to avoid ‘*unnecessary worry*’ (P8, FG2). However, others did not necessarily want to be given all of the information about their risk, with one participant stating that statistics such as the 5‐year survival probability would have ‘*scared them to death*’ and acknowledged contradictory feelings; she wanted both to be ‘*educated*’ and to ‘*be a bit oblivious*’ (P6, FG1).

For others, if they had not understood information they were given, they had searched the internet for answers. The consensus was, however, that this is a risky way to get information, as it could ‘*take you to some scary places in terms of other people's experiences*’ (P5, FG2). Several participants spoke favourably of charities (including KCUK and Macmillian) as a resource for providing access to needed additional information.

### A Need for Emotional Support

The emotional upheaval of a diagnosis of kidney cancer, surgery and subsequent recovery was widely acknowledged by the focus group participants. The period directly after surgery was especially difficult, as was learning to live with the ever‐present worry of recurrent disease. Some participants felt reassured by the knowledge that there are a range of treatment options available for recurrent disease. Others preferred to think as little as possible about the disease and their risk, and ‘*deal with it when I get the results*’ (P8, FG2). Another participant had not considered their risk of recurrence because their consultant had told them ‘*I got it all, don't worry about it*’ (P6, FG1).

These challenges were exacerbated for some people by additional worries, such as about their financial situation if they had been forced to reduce or stop working due to ill health. Several participants were also concerned about the wider impact a diagnosis of kidney cancer had on those close to them, including the emotional impact and the potential genetic risk of kidney cancer for their children.

Several participants spoke highly of charities that had provided them with resources, including counselling and access to support groups that had been ‘*a lifeline*’ (P5, FG1) since their diagnosis.

## Discussion

This study is the first to investigate in depth the patient experience of follow‐up care after surgery for kidney cancer. Through discussion with 14 individuals, living in the United Kingdom, who had previously had surgery for kidney cancer, we have identified a range of areas in which current care could be improved. In particular, while most participants felt that their physical needs had been met, many had struggled with unmet emotional needs and a lack of information and resources. This was especially noted in the period immediately following surgery, when feelings of abandonment were common, and around follow‐up scans and routine appointments, which were a source of anxiety. Our participants also described concerns about the lack of consistency among different hospitals around the United Kingdom, with differences in the content and quality of follow‐up care.

The transition from active treatment to follow‐up care has previously been identified as a difficult period for cancer patients in which they often experience feelings of distress [12] and abandonment [9]. In a UK‐based study, prostate cancer patients felt a need for more intensive support in the first year of follow‐up care, given their perceived vulnerability and anxiety [10].

Further, previous studies have found that patients felt unprepared for the transition to follow‐up care, due to insufficient information [9, 13, 14] or feeling overwhelmed by large amounts of information in a short period of time [14, 15]. A recent international survey of patients with kidney cancer found a lack of understanding around diagnosis and treatment decisions; 38% of respondents reported not being told their subtype of cancer and 25% had no understanding of their likelihood of survival [16].

A systematic review found that patients in multiple countries with a range of cancer types had unmet information needs [11]. The information needed to reassure patients, however, will vary by cancer type. For example, a study in prostate cancer found patients wanted more information about managing side effects [10], while in this study, participants identified kidney cancer‐specific topics, including ‘living with one kidney’ and risk‐stratified follow‐up, as areas with poor information coverage.

Concerns that follow‐up care varies among treatment centres has previously been identified by patients of multiple cancer types [10, 13]. While some studies have found that patients wanted a more ‘flexible’ or ‘holistic’ approach to follow‐up care [15, 17], this was not reflected in the views of the participants in our study, who were more concerned that practices and information should be standardized.

As seen among the participants of this study, fear of recurrence has previously been identified as a key challenge for patients, with 50% of patients anxious about recurrence in a recent survey [16]. Fear of recurrence has measurable effects on mental health after cancer treatment [18], although regular and ongoing surveillance can provide reassurance [11]. However, a recent survey of kidney cancer patients in the United Kingdom found that routine imaging appointments were a source of anxiety [7]. This was supported by the findings of our study, with anxiety around appointments and results affecting almost all of the participants, linked to uncertainty about scheduling and long waits for results.

### Recommendations for Changes to Care

Together, our findings suggest that the follow‐up care experience for many patients could be improved. Based on the experiences of the participants, we have developed a list of recommendations to address some of the challenges described, through relatively minor changes to the care pathway (Table 3).

**Table 3:** Recommendations for best practice.

	Stage(s) of follow‐up care	Areas in which patients identified a need for improvement	Recommendation
1	Immediately post‐surgery	A lack of information about aftercare post‐surgery	Patients should be provided with clear information about what to expect after surgery, including details of any wound care and strategies for pain management. This information should be delivered in the pre‐surgical clinic and reinforced prior to discharge from hospital
2	Immediately post‐surgery	A lack of information about living with one kidney	Patients should be provided with clear information immediately after surgery (such as a leaflet) on ‘living with one kidney’, including guidelines around alcohol consumption and medications to avoid (e.g., ibuprofen)
3	Initial follow‐up appointment	Not all patients have a good understanding of their follow‐up plan and the clinical justification for this in the context of their individual risk of recurrence Further, the explanation of a follow‐up care plan and the justification for this can be overwhelming and difficult to remember for patients—especially if this is delivered at the same time as their cancer diagnosis	Patients should be sent a letter after their first appointment after surgery (or given equivalent written information within the appointment), clearly outlining their personalized follow‐up care plan and the justification for this imaging schedule with respect to their individual risk
4	Initial follow‐up appointment Routine follow‐up care	Unexpected phone calls, or waiting for an unscheduled call, can be a source of anxiety and patients are likely to feel be less prepared for the appointment	Where remote appointments are used, for example, to give scan results, these should be scheduled with patients ahead of time, so that they have a chance to prepare and to minimize anxiety
5	Routine follow‐up care	Often, questions about follow‐up care—and similarly at other points in the care pathway—will not occur to patients until after an appointment	Patients should have an assigned point of contact (such as a clinical nurse specialist), who they can contact with questions that may arise outside of formal appointments
6	Routine follow‐up care	Anxiety around the scheduling of follow‐up appointments and waiting for scans is exacerbated by vague or flexible timelines	Care should be taken to manage the expectations of patients about when appointments will be scheduled, such as by giving windows (e.g., you will be invited for a follow‐up scan between 6 to 8 months from now) and realistic timeframes (e.g., you will get the results from your scan within 6 weeks). Ideally, scans and appointments (including the delivery of scan results) would be scheduled well in advance to minimize anxiety
7	Routine follow‐up care	Not all patients had the opportunity to see their follow‐up scans.	All patients should be offered the opportunity to see either their scan or their scan report. Where follow‐up appointments are remote, this should be sent to patients who ask for this information soon after the appointment (*via* email or letter as appropriate).
8	All stages	Many patients require additional emotional support after a kidney cancer diagnosis and surgery.	Clinicians should be mindful of the need for emotional support and provide signposting to appropriate resources (such as charities that provide counselling to cancer patients) to all patients. This information should be made available in the first follow‐up appointment and returned to as necessary at subsequent appointments.
9	All stages	Information needs are highly individual and sensitive.	Clinicians carrying out discussions about follow‐up care should take the lead from patients on how much information they want ‐ especially about the risk of recurrent disease. All patients, even those at relatively low risk, should be prepared for the possibility of recurrence.
10	All stages	Concerns that transitions (between departments or from secondary care to primary care) were often slow and resulted in information loss.	Relevant information about patients should be promptly sent to receiving departments and regularly sent to their primary care provider. Patients should be told how their information is being shared and if any changes are made.

Several of these recommendations (#1, #2, #3) include improving direct provision of information to patients, covering aspects of their follow‐up care (aftercare, life with one kidney, how follow‐up care is determined). Participants indicated that receiving this information directly from a clinician involved with their care, followed by a written explanation, would maximize recall and understanding. Two other recommendations cover the way in which information about follow‐up care is communicated (#5, #6), highlighting the need for patients to have a named individual or team to whom they can direct questions after their appointment and that patient anxiety around follow‐up appointment scheduling may be lessened by clear and realistic projections of the timetable. Good information provision may include signposting to resources where high‐quality and reliable information can be found, or as in one recommendation (#8), where support services (such as specialist counselling) not provided as part of routine care are available.

It also became clear in discussion with the participants that information needs are highly individual and sensitive. This creates challenges in making the delivery of this part of the care pathway suitable for all patients. We recommend that clinicians carrying out discussions about follow‐up care take the lead from patients on how much information they want, especially with respect to their risk of recurrent disease (#9). It has been shown in other studies, that risk recall is often poor [19], so it is important that patients are given both verbal and written explanations, and that they have the opportunity to return to the topic in subsequent appointments. However, it is also important that all patients, even those at relatively low risk, are prepared for the possibility of recurrence, for example, by emphasizing that low risk does not mean no risk.

Our recommendations also cover some aspects of the logistics follow‐up care delivery. Study participants identified unscheduled telephone appointments as a source of anxiety (if waiting for a call for several weeks) and alarm (if not expecting a call). We suggest, therefore, that all appointments are scheduled in advance (#4). Participants also indicated that scheduling scans and appointments well in advance (e.g., during the previous appointment) helped manage anxiety about the follow‐up care timetable (#6). We also recommend that, where possible, patients are given the opportunity to see their scan results at their follow‐up appointments (#7), as participants who had been offered this felt it had been helpful. Given the concerns expressed about delays and information loss when information is transferred between departments, we recommend that transfers of relevant information to other departments and primary care providers should be rapid, regular and transparent to patients (#10).

Other issues identified by patients with respect to their follow‐up care may require more substantial changes. For example, patients were concerned that risk‐stratified follow‐up care was not being applied consistently around the country. This, at least in part, reflects the current lack of certainty in the guidelines [3, 4], given that evidence for the benefits of risk‐stratified follow‐up care for kidney cancer is weak and no specific recommendation for stratification method is recommended.

### Strengths and Limitations

To our knowledge, this is the first study to explore patient experience of kidney cancer follow‐up in depth. We recruited patients from across the United Kingdom, including a mixture of men and women and a range of ages. However, our method of recruitment—via a social media callout—may have introduced some selection bias. Our study participants were relatively young (72% of participants in this study were aged under 60 years, although more than 60% of kidney cancer diagnoses occur in the over 65s [20]) and surgery was relatively recent for many of the participants (43% within 1 year and 79% within the last 2 years). It is likely that patients with stronger views about their follow‐up care took part in this study, however, a range of different experiences (both positive and negative), diagnoses and prognoses, were represented. The method of recruitment means that it is not possible to determine the response rate, as we do not know how many eligible people saw the advert.

Given the timing of the focus groups (January 2022), many of those recruited had received their cancer treatment and initial follow‐up during the COVID‐19 pandemic, which may have influenced their perspective on the care they received. However, in discussion in the focus groups, participants expressed understanding of the unique pressures on the healthcare system due to COVID‐19 ‘*I get there's a*…*lot going on a moment, so trying to stick to those expectations…is really challenging*.’ (P5, FG2) and similar topics were raised by participants who began follow‐up care before and after 2020.

### Conclusions

We explored patient experiences of follow‐up care after kidney cancer in focus groups. We identified six themes that described current patient experiences (*feelings of abandonment, uncertainty about the plan, anxiety about appointments, variation in care, a need for information and a need for emotional support*) and used these to form a series of recommendations for best practice of follow‐up care after kidney cancer surgery.

## Disclosure of Interests

Grant D. Stewart has received educational grants from Pfizer, AstraZeneca and Intuitive Surgical, consultancy fees from Pfizer, Merck, EUSA Pharma and CMR Surgical, travel expenses from Pfizer and speaker fees from Pfizer.

## Funding

This work was funded by Kidney Cancer UK (REDY.GAAA). Hannah Harrison is supported by an International Alliance for Cancer Early Detection Project Award (ACEDFR3_0620I135PR007). Grant D. Stewart's work on this topic is funded by Kidney Cancer UK, the Urology Foundation, the Rosetrees Trust, Yorkshire Cancer Research and Cancer Research UK and supported by the Mark Foundation for Cancer Research, the Cancer Research UK Cambridge Centre [C9685/A25177] and National Institute of Health Research (NIHR) Cambridge BRC. Juliet Usher‐Smith is supported by an NIHR Advanced Fellowship (NIHR300861). The views expressed are those of the author(s) and not necessarily those of the NIHR, NHSBT or the Department of Health and Social Care.

## Supporting information


**Appendix S1** Focus group advert
**Appendix S2** Participant information sheet
**Appendix S3** Participant questionnaire
**Appendix S4** Focus group topic guide
**Appendix S5** Final coding framework with illustrative quotations
