# Supplementary material for: Patient experience of follow‐up after surgery for kidney cancer: a focus group study
Source: BJU Int. 2023 Feb 22;132(1):47–55. doi: 10.1111/bju.15982 (PMC10952230; doi:10.1111/bju.15982)
Supplement: Supplementary file 1 — Appendix S1 Focus group advert Appendix S2 Participant information sheet Appendix S3 Participant questionnaire Appendix S4 Focus group topic guide Appendix S5 Final coding framework with illustrative quotations [file BJU-132-47-s001.pdf]

Focus Group Advert:

Kidney Cancer UK working with researchers at the University of Cambridge would like to engage with patients who have had surgery for kidney cancer to discuss their experiences in an online focus group.

This research project aims to explore patient perspectives of follow-up care and surveillance after kidney cancer surgery. In small group sessions, we will discuss experiences and reflections on current follow-up care, addressing topics including decision-making, communication of follow-up schedules and the management of follow-up appointments. We are keen to work with patients who have had both positive and negative experiences of follow-up care.

We are looking forward to working with patients to better understand their views on this topic. We plan to hold online focus groups between the 25th of January and the 3rd of February.

If you are interested in being part of this focus group and important piece of research, email <contact details for KCUK here> with your details and we will contact you.

UNIVERSITY OF CAMBRIDGE
Public Health and Primary Care
The Primary Care Unit

Kidney Cancer UK
The UK's leading kidney cancer charity for over 20 years

Exploring the Patient Perspective of Follow-up Care after Surgery for Kidney Cancer: a Focus Group Study

Participant Information Sheet

Thank you for considering taking part in our focus group study to explore patient views on follow-up care after kidney cancer surgery.

Please take time to read this information carefully to understand why the research is being done and what it will involve for you.

Who is organising the research?
The study is being led by doctors and researchers at the University of Cambridge. It is funded by Kidney Cancer UK and has been reviewed by the University of Cambridge Psychology Research Ethics Committee.

What is the purpose of the study?
The aim of this study is to explore patient views of current follow-up care and surveillance (e.g. imaging) after kidney cancer surgery. In this research project we will explore the experiences of patients during follow-up and bring together evidence that could be used to justify changes to current care.

We are asking people to take part in an online focus group. This is where a group of members of the public are brought together to talk about a specific topic. Through this we hope to understand patient perspectives, both through hearing about individual experiences and group discussion. A researcher will also participate in the group and chair the discussion.

Who is taking part?
We are recruiting 4-8 people to each focus group. Only people over the age of 18 who have had surgery for kidney cancer will be invited to participate. All participants will be recruited via a social media callout by Kidney Cancer UK, and the sessions will be organised by researchers at the University of Cambridge.

Do I have to take part?
Your involvement in this study is entirely voluntary and you can leave at any time without giving a reason and there will be no consequences for doing so. If you choose to withdraw after the start of the focus group, it may not be possible to delete all of the information you have provided due to difficulty in identifying individual participants' comments in the group discussion.

What happens next?
Before joining the session, you will asked to fill in a form indicating that you consent to participate in this research study and also to fill in a short questionnaire. If you are not able to complete these electronically (using a word processing package) a member of the research team will be happy to go though them with you via Zoom.

The focus groups will begin with introductions and then all participants will be invited to share their intial thoughts on follow-up care. We will then spend time discussing

topics such as decision making, communication and scan appointments. The discussion will be chaired by a member of the research team.

The focus group will be conducted online using Zoom video conferencing. We will encourage you to have your video turned on but will not insist on it. All the discussions as well as the group decision and reasons will be video recorded using Zoom. The recordings will then be transcribed by an external company who will have signed a confidentiality agreement.

How much time will it take?
Each focus group will last about one hour duration.

What are the possible benefits of taking part?
By taking part in this study, you will have the opportunity to share your experiences of follow-up care after kidney cancer surgery and hear from others with similar experiences. It is also an opportunity to help influence follow-up care in the future by sharing your views and opinions.

Are there any disadvantages or risks of taking part?
Discussion experiences of cancer, including diagnosis, treatment and on-going care, can be a sensitive topic. If you become upset by anything during the focus group, you may exit at any stage and there are no consequences associated with doing so.

If discussing these topics causes worry or anxiety, or if you have questions about your own care, there are resources avaliable to support you.

- You can contact Kidney Cancer Careline (https://www.kcuk.org.uk/kidney-cancer-care, 0800 002 9002), a helpline that is operated by a Health Professional team that provides both clinical and psychological support for kidney cancer patients 7 days a week (9pm-4pm on weekdays).
- You can also contact Kidney Cancer UK Counselling (https://www.kcuk.org.uk/kidney-cancer-counselling, 0300 102 0101) a service that provides free one-to-one counselling for patients over the phone with a qualified counsellor.
- You can also contact the Macmillan Support Line (0808 808 0000), a helpline that provides support and information to people living with cancer 7 days a week (8am-8pm everyday).

If you are in need of immediate support during this session, please send a message to Dr Usher-Smith, who is a member of the research team and a GP, using the private chat function and she will assist you.

The focus groups are not medical assessments and the research team will not be able to provide any medical advice. Any clinical concerns should be addressed with your GP or your Cancer Specialist Team.

Will I be paid for taking part?
No, your participation is entirely voluntary, and you will receive no payments or other benefits for taking part.
Will my taking part in this study be kept confidential?
All information that is collected from you during the course of the study is anonymised and stored securely.

The University of Cambridge is the sponsor for this study based in the United Kingdom. They will be using information from you in order to undertake this study and will act as the data controller for this study. This means that they are responsible for looking after your information and using it properly. The sponsor organisation will keep identifiable information about you for 12 months after the study has finished to ensure your safety and allow the study to be reviewed by the authorities after it is finished.

Your rights to access, change or move your information are limited, as the sponsor organisations need to manage your information in specific ways in order for the research to be reliable and accurate. To safeguard your rights, we will use the minimum personally-identifiable information possible.

To find out more about how the sponsor uses your information, please visit: www.medschl.cam.ac.uk/research/information-governance, or email the Information Governance team at: researchgovernance@medschl.cam.ac.uk.

What will happen to the results of the study?
This research is important for informing decision makers during the development of health policies. All findings will be published in peer-reviewed scientific journals and presented at national and international conferences. Anonymous direct quotations from the study may be used but you will not be identifiable in any of these. We can provide a summary of the results for you as well as share the completed publications with you. Anonymous information from the study may also be looked at by responsible individuals from the sponsor, regulatory authorities and research personnel, and used to support other research in the future.

If you provide additional consent, audio clips from the recordings may be used when presenting the results. All audio clips will be edited to remove identifiable information and each clip that includes your voice will be sent to you, for you to hear and agree to its use. If you do not want audio clips used you can still participate in the study.

What if there is a problem or something goes wrong?
If you have any concerns or complaints about any aspects of the study, you can telephone the study lead, Dr Juliet Usher-Smith, on 01223 748693 or email her at: jau20@medschl.cam.ac.uk.

Alternatively, if you would like to write to her, please send your letter to:

Dr Juliet Usher-Smith
c/o Urological Malignancies,
CRUK Cambridge Centre, Cancer Research UK Cambridge Institute,
University of Cambridge
Li Ka Shing Centre, Robinson Way,
Cambridge, CB2 0RE

We will reply to your email or letter in writing, unless you enclose your telephone number and wish to discuss your concerns by telephone.

Thank you very much for reading this information leaflet and choosing to take part in this study

UNIVERSITY OF CAMBRIDGE
Public Health and Primary Care
The Primary Care Unit

Kidney Cancer UK
The UK's leading kidney cancer charity for over 20 years

Exploring the Patient Perspective of Follow-up Care after Surgery for Kidney Cancer: a Focus Group Study
Questionnaire

This questionnaire is intended for people participating in one of the focus groups. We would like to ask you a few questions about yourself. This will help us to describe the people recruited for the focus groups when we present the results of this research study.

Participant ID: XXXXXXXXX

How old are you?

- ☐ <40
- ☐ 40-49
- ☐ 50-59
- ☐ 60-69
- ☐ 70-79
- ☐ 80-89
- ☐ >90
- ☐ Prefer not to say

What is your gender?

- ☐ Woman
- ☐ Man
- ☐ Neither
- ☐ Prefer not to say

What is your ethnic group?

- ☐ White
- ☐ Mixed/Multiple ethnic group
- ☐ Asian/Asian British
- ☐ Black/African/Caribbean/Black British
- ☐ Other, please describe______
- ☐ Prefer not to say

In what region of the UK do you live (or have most recently lived)?

- ☐ Scotland
- ☐ Wales
- ☐ Northern Ireland
- ☐ England, South East
- ☐ England, London
- ☐ England, North West
- ☐ England, East
- ☐ England, West Midlands
- ☐ England, South West
- ☐ England, Yorkshire and the Humber
- ☐ England, East Midlands
- ☐ England, North East
- ☐ I have never lived in the UK
- ☐ Prefer not to say

How long ago were you first diagnosed with kidney cancer?

- ☐ <1 year ago
- ☐ 1-2 years ago
- ☐ 2-5 years ago
- ☐ >5 years ago
- ☐ Prefer not to say

Have you attended follow-up appointments since having surgery for kidney cancer?

- ☐ Yes
- ☐ No
- ☐ Prefer not to say

Focus group guide: patient experiences of kidney cancer follow-up

Welcome
HH: "Hi, my name is… and I am joined by …, my colleague … and a practising GP. "

HH: Take consent to record the session and start recording.

HH: "We anticipate the discussion lasting approximately one hour. During discussion, it would be great if you could indicate (raise hand or wave) if you want to contribute and I will ask you each to contribute in turn. You may wish to mute your microphone when others are talking."

"We are aware that some of the topics we are planning to discuss today may be sensitive and challenging. We remind you that you are free to withdraw at any time. Resources and organisations able to support you (including the Kidney Cancer Careline and Kidney Cancer UK Counselling) are indicated on the participant information sheet that was sent to you and we will also post them in the zoom chat."

JUS: Post details of Kidney Cancer UK Counselling, Kidney Cancer Careline and JUS's telephone number in the public chat.

HH: "If you are in need of immediate support during this session, please send a message to Dr Usher-Smith, who is a member of the research team and a GP, using the private chat function and she will assist you."

Aim of today's session: To explore your views on follow-up care after kidney cancer surgery

Introductions

Ask participants to introduce themselves and share why they decided to participate in this focus group.

Questions and Discussion

First thoughts

- After having kidney cancer surgery, how did you feel about your follow-up plan?
- Were you made aware of your full follow-up schedule and how it was determined?
- What are your views on how this follow-up plan was presented to you?
- If you have been to some follow-up appointments, have your views on follow-up changed over time?

At the moment, decisions about the intensity of follow-up after kidney cancer surgery are made based on a risk estimate that takes different properties of the initial cancer into account (stage, size, aggressiveness etc.).

- What do you think about intensity of follow-up being determined by factors relating directly to the cancer?
- How do you think this decision-making process should be presented to patients?
- What information about this process would you like to have available (during or after consultation about follow-up)?

Over long follow-up periods (>5 years) other factors – including age and broader health concerns – become important. For example, longer follow-up schedules may be more appropriate for people diagnosed with kidney cancer at a younger age. In the future, factors such as age, sex and other health conditions could be included in the risk assessment used to determine follow-up schedules. This may result in more intensive follow-up for some people, but less follow-up for others.

- What do you think about including additional factors such as age or other health concerns?
- Would it be acceptable to decrease follow-up intensity for people assessed to be at lower risk?

We are also interested in the process of attending follow-up appointments and how this works in practice:

- Did you have a clear idea of what a follow-up appointment would be like?
- If you have attended a follow-up appointment, how did this go in practise?
- How do you think the results of tests (e.g. scans) should be communicated?

We are also interested in the overall impact of current follow-up (specifically imaging) on patients and the healthcare system. *If not mentioned, specifically ask about **worry about results** and **radiation exposure from imaging**.*

- What do you think are the benefits or harms of current follow-up to patients?
- How do you feel about the frequency of the imaging you have received?
- Do you think it would be acceptable to reduce or increase the amount of imaging provided in follow-up?

Final thoughts

- Do you have any final thoughts on this topic?
- **Do you have any concerns that we have not yet addressed?**
- Is there anything we have not asked that you think would be useful?

Wrap-up and Closure

Many thanks for sharing your time and experiences of this difficult topic. If any concerns have arisen for you during this session, we will be available to talk with you immediately afterwards. We also want to remind you of the available resources should you feel anxious or worried following this session.

Repost details of Kidney Cancer UK Counselling and Kidney Cancer Careline in the chat.

Thank you

Appendix 5

Key Themes: Abandonment, Uncertainty about the plan, Anxiety about follow-up, Variation in Care, Need for information, Unmet emotional needs

Other important areas: Impact of the pandemic

Themes	Distinct Codes	# Sections Labelled with this Code	Illustrative Quotations (T – Tuesday focus group, W – Wednesday focus group)
Abandonment (lack of support post-surgery)	Lack of consistency	23	"I think that's a big thing for a lot of people is that transfer from Urology to Oncology, I think that's where some stuff falls down" (T4)
	Feeling Abandoned	31	"at the time I just sort of felt sort of tossed into the wilderness after my operation" (T3)<br>"My problem was after my treatment stopped and I never heard a thing from anybody" (T5)<br>"And I wanted to be part of this because although the surgery went really well I do feel as I have been left a little bit to my devices." (W4)<br>"And then I didn't hear anything, nothing at all. Nothing. Completely into a black hole." (W6)
	Lack of Pain Management	8	"I've been left in a lot of pain and very little support or follow up; so it sounds a very similar story." (W2)<br>"Just 23 hours after the op out you go not even given a bottle of paracetamol." (W5)
Uncertainty about the plan	Discussions about risk	32	"And then there was a plan that you would have three monthly after that because you were in the high risk category…. And really understanding the reason why it was three months and no longer or quicker than that as well; that was really well explained to me." (T4)<br>"I guess I received a letter explaining but then some of the terminology in the letter I just didn't understand, and nobody really explained it to me even when I asked initially." (W8)<br>"He said to me the likelihood was being very high risk we would reckon 85% of people would see some reoccurrence of cancer somewhere else in the body in the next five years. But he just threw that out there and it was... You don't have time to react" (T4)<br>"If they are going to use the Leibovich score then everyone uses the Leibovich score. If some are and some aren't then they shouldn't use it at all, they shouldn't give it to people." (T4)
	Understanding decision making	17	"Mine was clearly explained to me on the phone and in a letter exactly what was we were going to be doing for the next two years and he said it was because it was a particularly aggressive cancer, so they did explain all of that to me on the phone and in a letter as well." (W4)<br>""So we've moved quite quickly from every three months to every six months and I am always a bit like at what stage do you understand why it's the difference in time? Because when you talk to people and you are like 'Well I am being seen quicker' and is that because I am higher risk?" (T6)
	The end of follow-up	7	"I must admit when it said it was the five year survival rate it felt there was no plan for after five years and nobody mentioned that... " (W4)

Anxiety and follow-up	Planning appointments	26	"I always had to chase the fact that I was going to have a follow up scan..." (T3)<br>"…when I leave my consultant he books his next appointment with me straightaway. So within two days of coming home I know when I am seeing him ..." (T6)<br>"And then when they said they would speak to me within six weeks, when it got to the sixth week they cancelled my appointment and said I have to wait another six weeks so you can imagine how anxious..." (W8)<br>"I think the thing I found hardest with all of it was just that expectation management, so if they said six months and it is seven I would find that really difficult." (W5)
	Influence of diagnostic experiences	22	"..that <a kidney mass> should be picked up in my follow ups, I said 'But they didn't pick it up in an ultrasound in the first place'." (T1)<br>"One of the questions we asked 'Is how long it had been there for?' And he reckoned it had been growing on my kidney for five years, five years with no symptoms. And it is one of the scariest things that I've ever heard…" (T4)
	Waiting for results	57	"... and I always had to chase the results as well. Because obviously you suffer from 'scanxiety' don't you once you've had a scan. So waiting for the results was horrendous" (T3)<br>"... For me personally I think it is one of the worst things is just waiting for those results all the time..." (T3)<br>"And for the two weeks or three weeks afterwards you are left thinking any time that phone rings it is going to be an oncologist telling me it is back." (T4)<br>"This time I've actually got the call for the CT scan a month before I am due to see him so it is a long time to wait and it does make you... because you are constantly like 'Oh gosh I am just waiting, waiting, waiting'." (T6)<br>"So when I've had a scan I don't worry about the scan at all because I normally.... if I have a scan I get results within two weeks and that's always been the case and if it is bad they ring you…" (W7)<br>"And it is the same with the results, you say we will find out in three weeks and it takes six that's really hard to deal with. If originally they had said six I wouldn't have been happy but at least I would have had that expectation managed." (W5)
Variation in Care	Postcode lottery	22	"…because there's a real disparity from one end of the United Kingdom to the other in terms of what we get as after care.." (T4)<br>"My after care was actually quite good and from reading what other people have put I think of lot of it is postcode." (T5)

			"It should be standard NHS; you have had kidney cancer you've had a partial or you've had a radical here's the fact sheet." (W2)
	Lack of Kidney Cancer Expertise	9	"I think if you sort of live under one of the like specialist kidney cancer places you get a lot more information and a lot more help and things. If you sort of live somewhere that is not I think... you are very much on your own to your extent a lot of the time because... well because of the way it is.." (T3)
Need for information	Information immediately post-surgery	32	"So I just felt very much just right you are home and that's it and there is no advice or anything for you." (T3)<br>"I didn't even get any idea of how to... what to do with the stitches or anything like that." (WX)<br>"..so they said 'Drink lots of water but not too much', and that was kind of it. Then some people said 'Don't use Ibuprofen' but that's what I had in the hospital and I never really got a clear kind of living with one kidney guidance." (W5)<br>"How do you live with one kidney? I didn't really get too much explanation it has been a bit hit and miss….And I've just had to do my own research and try and work out what is there." (W3)<br>"And I didn't know I had to inject myself every night as well with anticoagulants which was... after surgery for the next month, so I found that quite difficult and unexpected." (W8)
	Experiences of first follow-up appointment	33	"…it was a telephone call that wasn't pre-planned it came out of the blue. The phone started ringing one day and it was my surgeon who said 'I have got your histology results are you sitting down? Are you ready to hear what it is?'" (T4)<br>"At my six week appointment when I was told everything that it was cancer and that I would have annual scans he mentioned ultrasound or it might be CT. So I sort of thought okay at the time I was so blown away that it was cancer so it was hard to take it in…" (T1)<br>"But the main follow up was a four week call with the surgeon to explain what they had found, which they followed up with a letter with a care plan in explaining when the scans would be for the six monthly, then yearly, then two yearly for the next ten years. So they explained all that to me quite well." (W1)
	Charities as a resource	28	"But the Kidney Cancer Facebook page, the group all those types of things have all been fantastic as well as a real library of knowledge; there's nothing there than people have been through things themselves.." (T4)

			“So when I saw that in the letter I went ‘What on earth does this mean?’, straight on the phone to Kidney Cancer saying ‘What does this mean?’ And Kidney Cancer were able to tell me what it was, what each individual thing meant and what that meant for me going forward.” (T4)
	More or less information?	20	“I am always saying ‘Can I see the scans?’ because I want to see what it looks like but some people might not want to do that.” (T3)<br><br>“I think I would have been scared to death if he <the consultant> had told me 85% chance in the next five years... I want to be educated but at the same time I want to be a bit oblivious.” (T6)<br><br>“I think you know if they are going to give you this information it has to be explained at the time because it does cause a lot of anxiety and a lot of unnecessary worry…I’m an adult so you need to treat me like an adult and not just kind of throw science at me and then not explain.” (W8)<br><br>“I’ve just Googled stuff which is obviously good in some ways but it can take you to some very scary places in terms of other people’s experiences..” (W5)
Emotional support	Learning to live with risk	20	“…take it a month at a time, a step at a time.” (W6)<br>“for me at the moment I’m trying to get on with things to keep busy and to block it out of my mind and just be positive and just deal with it when I get the results…” (W8)<br>“I think support is mainly emotional I don’t think it’s physical that we deal with. Emotional, psychological support that is important, perhaps that is just me I don’t know.” (W2)
	The wider impact	6	“..I wanted to make sure that there wasn’t a genetic one <association> because having kids I wanted to make sure there was nothing that would eventually could be passed on to my kids.” (T4)<br>“It would be nice to go back to work <I’m> worse off after the operation than I was before.” (W2)
	Struggling after surgery	6	“I have had a mixture of emotions where I’ve been at a very dark place and there are days when I’m very positive.” (W3)
The impact of the pandemic	Mention of covid	19	“We went into lockdown about a couple of weeks after I had my surgery so that was a telephone call, and it was a telephone call that wasn’t pre-planned it came out of the blue.” (T4)<br><br>“But again it was me chasing them and quite honestly with the Covid going on at the moment and the hospitals I am not really expecting to hear anything for a while.” (T1)

			“I actually was really pleased that I was forced to go on my own <to an appointment> … I was able to ask all the questions I wanted to ask without someone interjecting and their emotions and their feelings.” (T6)
